# Behavioral Compensations and Neuronal Remodeling in a Rodent Model of Chronic Intervertebral Disc Degeneration

**DOI:** 10.1038/s41598-019-39657-6

**Published:** 2019-03-06

**Authors:** Elizabeth M. Leimer, Matthew G. Gayoso, Liufang Jing, Simon Y. Tang, Munish C. Gupta, Lori A. Setton

**Affiliations:** 1 Department of Biomedical Engineering, Washington University in St. Louis, One Brookings Drive, Whitaker Hall, Suite 190, St. Louis, MO 63130 USA; 2 Department of Orthopedic Surgery, Washington University in St. Louis School of Medicine, 425 S Euclid, Suite 11627, St. Louis, MO 63110 USA; 3 Department of Biomedical Engineering, Duke University, 101 Science Drive, Fitzpatrick Center, Room 1427, Durham, NC 27708 USA; 4 Albany Medical College, 43 New Scotland Ave, Albany, NY 12208 USA

## Abstract

Low back pain is associated with degeneration of the intervertebral disc, but specific mechanisms of pain generation in this pathology remain unknown. Sensory afferent nerve fiber growth into the intervertebral disc after injury-induced inflammation may contribute to discogenic pain. We describe a clinically relevant behavioral phenotype in a rodent model of chronic intervertebral disc degeneration which provides a means to map sensory neuron changes to a single affected lumbar intervertebral disc. Unilateral disc puncture of one lumbar intervertebral disc revealed a bilateral behavioral phenotype characterized by gait changes and decreased activity. Moreover, neurons extracted from the dorsal root ganglia in animals with intervertebral disc injury demonstrated altered TRPV1 activation *in vitro* independent of exogenous NGF administration. Finally, neuronal nuclear hypertrophy and elevated expression of p75NTR provide evidence of active adaptation of innervating sensory neurons in chronic intervertebral disc degeneration. Therefore, this model and findings provide the template for future studies to establish specific mechanisms of nociceptive pain in chronic intervertebral disc degeneration.

## Introduction

Low back pain (LBP) affects up to 85% of the population in their lifetime^1^ and is the leading cause of disability worldwide, ranking sixth in contributions to the musculoskeletal burden of disease^2^. Low back pain is associated with degeneration of the intervertebral disc (IVD) in 40% of cases^3^, documented as changes on imaging modalities such as loss of hydration or loss of disc height using T2 MRI or plain radiographs^4,5^. The IVD is the largest avascular and aneural structure in the body and is characterized by low interstitial oxygen tension, which contributes to challenging conditions for cell survival in adult IVD tissue. With degeneration, the IVD exhibits reduced cellularity that further impairs the ability for self-repair, as well as decreased hydration, loss of interstitial fluid pressure, and changes in matrix composition including increased fibrosis and loss of proteoglycans^6,7^. No disease modifying interventions currently exist, and available treatments fall short of managing chronic low back pain or risk further complications. Specific mechanisms of pain generation in this pathology remain unknown, although there is evidence to support the hypothesis that sensory afferent nerve fiber growth into the IVD after injury-induced inflammation may contribute to discogenic pain^8^. This support is derived primarily from observations of nerve growth factor (NGF)-dependent nerve fibers present in degenerate human and animal IVD tissue^9,10^ compared to non-pathologic IVDs, where innervation is limited to the outer three to four lamellae of the anulus fibrosus (AF)^8^. However, the mechanistic involvement of these innervating sensory nerves in IVD degeneration and their contributions to clinical symptomatic low back pain remain unclear.

Lumbar disc puncture (LDP) is the most widely-accepted approach to induce IVD degeneration in animal models and has been shown to replicate the loss of IVD height and hydration, and decrease in cellularity observed clinically^11^. LDP models have also been found to induce altered gait parameters such as a trend toward increased percentage of stride in stance and reduced pressure hyperalgesia on the dorsal surface over the area of the injured IVDs in rodents, reflective of behavioral changes or pain-related sensitivity, but at the shortest timepoints after surgery (≤7 weeks)^12,13^. Importantly, LDP in animal models induces transient upregulation of pro-inflammatory cytokines in the IVD, including TNF-α and IL-1β, compared to more persistent upregulation which has been widely observed in human pathological samples^11,14^. These pro-inflammatory cytokines are known to induce regulators of pain, including NGF and substance P mRNA expression and protein secretion^15,16^. The transient inflammatory response induced by LDP injury in the rat resolves within 2 weeks; however, the neuropeptide NGF remains elevated in this model for as long as 8 weeks post-injury^14^ and may be a key mediator in inflammatory pain^17^.

Upregulated NGF synthesis sensitizes the innervating primary afferent neurons to produce hyperalgesia^18^ via upregulated tyrosine kinase A (TrkA) expression in small dorsal root ganglion (DRG) neurons^17^. Elevated NGF can also promote expression of substance P^18^, calcitonin gene-related peptide^17^, transient receptor potential cation channel subfamily vanilloid member 1 (TRPV1)^19^, and sodium ion channels that may lead to long-term adaptive effects on nociceptors^20^. Moreover, NGF binding to TrkA or p75 neurotrophin receptor (p75NTR) can initiate signaling to promote neuronal survival or additional inflammation^21^. NGF binding to TrkA also regulates collateral sprouting of sympathetic fibers to DRG neurons that may relate to maintenance of chronic pain^22^ and can acutely sensitize sensory neurons to capsaicin^19^. In this context, we hypothesized that molecular changes in the degenerated IVD following lumbar disc puncture could contribute to an adaptive sensory neuron response that may be active in chronic IVD degeneration and play a role in discogenic pain.

## Results

### LDP injury induces a distinct timeline of behavioral changes

First, we conducted a pilot study to determine the utility of commonly used behavioral assessments to detect changes in a LDP model of chronic IVD degeneration, as well as an overall timeline of behavioral changes in this model. Lumbar disc puncture (LDP) injury was achieved by repeated punctures (10x) of the lateral aspect of a single rat IVD (L5-L6) with a 27 gauge needle and injection of 0.3 mL air to disrupt the internal contents (Supplementary Fig. S1). Sham animals underwent surgery for exposure of the IVD with no induced damage. To determine the time course of model development after LDP, behavioral changes were assessed weekly until post-operative week 20 (Table 1). We found that the behavior of both sham and LDP groups deviated from that of naïve animals at post-operative week 1, which we attributed to surgical trauma and associated pain. Acute changes in static weight-bearing and mechanical sensitivity appeared to resolve by post-operative week 3 and 4, respectively, (Supplementary Fig. S2a,b) and showed a large amount of variability after this period. Gait parameters showed an overall trend toward resolution around post-operative week 5 (Supplementary Fig. S2c–e). Distinctly different changes in unilateral parameters (i.e., affecting only one side of the body) began at post-operative weeks 16 (Supplementary Fig. S2c,d) and 18 (Supplementary Fig. S2a), as well as bilateral changes at post-operative week 18 (Supplementary Fig. S2e). These results revealed a distinct timeline of behavioral changes after LDP injury: acute changes induced by surgical trauma resolved at 4–6 weeks post-surgery, a period resembling pre-surgical values was sustained from 6–16 weeks post-surgery, and changes were again observed at 16–20 weeks post-surgery. Based on this pilot data, the number of behavioral assessment timepoints was reduced for the subsequent study, and the resulting data were grouped into acute (0–4 weeks), intermediate (6–16 weeks), and chronic (18–20 weeks) time bins for analysis. A clear trend toward increased mechanical sensitivity was not observed after the initial post-operative period, leading us to conclude that the LDP model is not associated with a robust distal neuropathy phenotype. In full cohort testing, we added operant measures of overall activity, including open field, burrowing, and a lateral bending maze.

**Table 1:** Behavioral Assessments.

Assessment Type	Assessment	Parameters Measured
Bilateral	Open Field Arena	% Time spent traveling
	Burrowing	Weight burrowed (g)
	Site-specific pressure sensitivity	Applied pressure threshold
	Treadmill Gait	Forelimb vs hind limb stride frequency
	Lateral Bending Maze	Pain tolerance
Unilateral	Static Weight-Bearing	% Weight on operated side
	Treadmill Gait	Hind limb swing (s)
		Hind limb duty factor
	Mechanical Sensitivity	50% withdrawal threshold

### A bilateral behavioral phenotype develops after LDP injury

**Figure 1:**
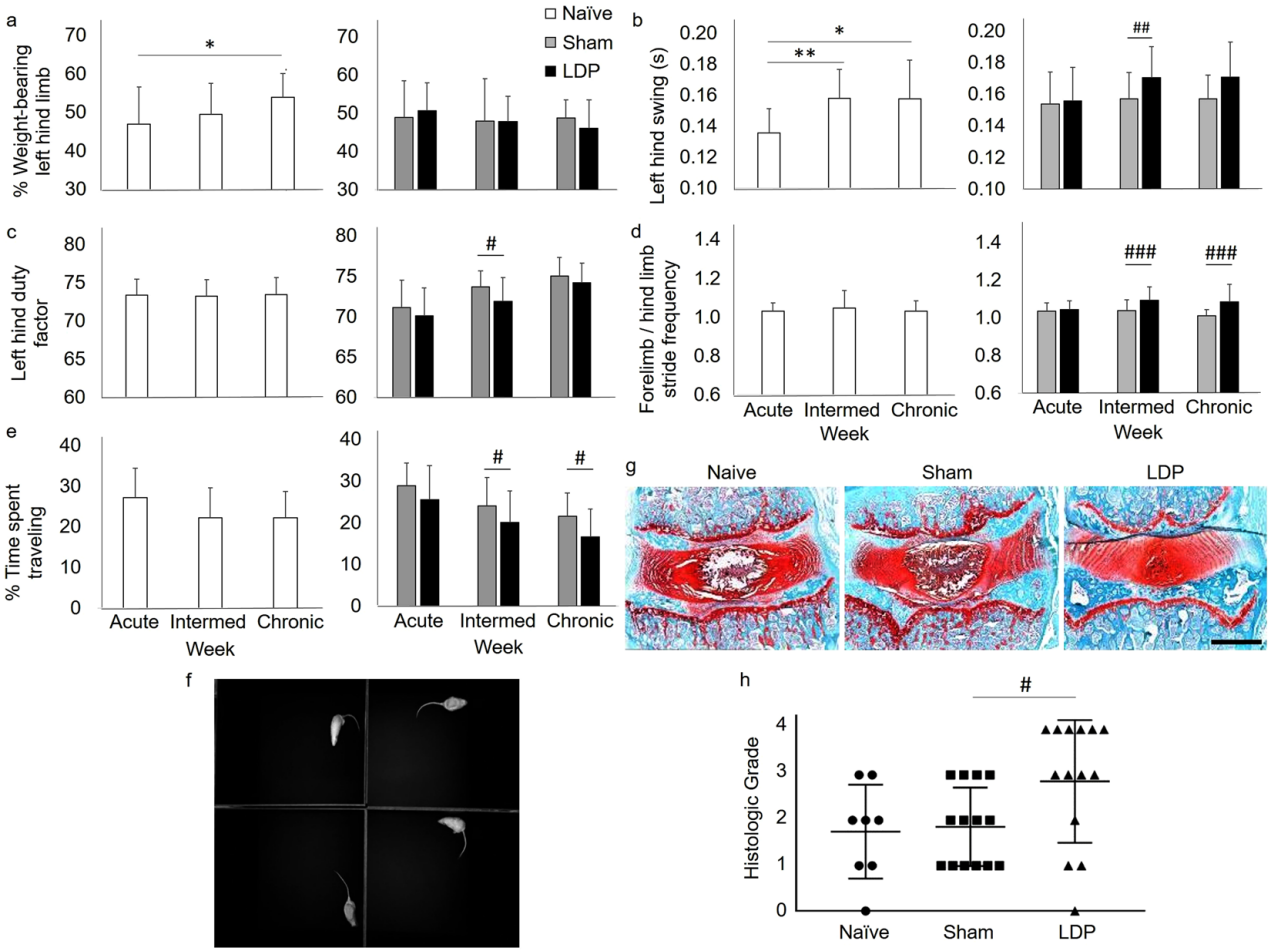
LDP injury induced a behavioral phenotype characterized by decreased movement. (**a**) Static weight-bearing for the left hind limb was shown to change over time in the naïve animals (1-way ANOVA with Tukey’s post-hoc test, *p < 0.05). No differences in this parameter were detected between surgical groups. (**b**) Swing duration for the left hind limb was shown to change over time in the naïve animals (1-way ANOVA with Tukey’s post-hoc test, *p < 0.05, **p < 0.01) and was also found to differ between sham and LDP groups at the intermediate time only (2-way ANOVA with Sidak’s post-hoc test, ^##^p < 0.01). (**c**) Left hind limb duty factor showed no changes over time in the naïve animals. This parameter was found to differ between sham and LDP groups at the intermediate time only (2-way ANOVA with Sidak’s post-hoc test, ^#^p < 0.05). (**d**) Forelimb vs hind limb stride frequency showed no changes over time in the naïve animals. This parameter was found to differ between sham and LDP groups at the intermediate and chronic times (2-way ANOVA with Sidak’s post-hoc test, ^###^p < 0.001). Effect size associated with statistical power is 1.1. (**e**) The percentage of time spent traveling showed no changes over time in the naïve animals. This parameter was found to differ between sham and LDP groups at the intermediate and chronic times (2-way ANOVA with Sidak’s post-hoc test, ^#^p < 0.05). Effect size associated with statistical power is 0.80. (**f**) Aerial view (camera view) of arenas during the open field assessment. (**g**) Representative frontal sections of rat L5-L6 IVDs. Scale bar: 1 mm. (**h**) Grade of degeneration for L5-L6 IVD was found to differ between sham and LDP groups (Wilcoxon rank sum test, ^#^p < 0.05). Effect size associated with statistical power is 0.88. All error bars represent standard deviation.

In full cohort testing of naïve, sham and LDP models, assessments revealed a behavioral phenotype for the LDP group characterized by bilateral functional changes and an absence of unilateral changes at the chronic timepoint. Unilateral changes for percent hind limb weight-bearing (Fig. 1a) as well as gait parameters of left hind limb swing duration (Fig. 1b) and duty factor (Fig. 1c) showed no differences between surgical groups of sham and LDP. However, the LDP group showed decreased hind limb stride frequency compared to that of the forelimbs (Fig. 1d; intermediate/chronic: p < 0.001) and decreased activity (Fig. 1e,f; intermediate: p = 0.049, chronic p = 0.044), both bilateral parameters which have been shown to change in models of inflammatory pain and spinal cord contusion^23,24^. No changes were detected for burrowing activity, site-specific pressure hyperalgesia, or pain tolerance on lateral bending (Supplementary Fig. S3). Degenerative histological changes (Supplementary Table S1) were present in the IVD of all groups harvested at the chronic timepoint (Fig. 1g), but more severe degeneration was found in IVDs of the LDP group (Fig. 1h; p = 0.019). Correlation analyses revealed no relationship between grade of IVD degeneration and activity or gait parameters at the chronic timepoint (Supplementary Table S2), suggesting a more complex relationship between onset of symptoms and degenerative IVD changes. Taken together, these results suggest that pain-related, bilateral phenotypic changes are associated with chronic IVD degeneration in this model. We subsequently investigated NGF-related changes in neurons from DRGs known to innervate the L5-L6 rat IVD^17^ in this model of chronic IVD degeneration.

### TRPV1+ DRG neurons are functionally different in painful IVD degeneration

**Figure 2:**
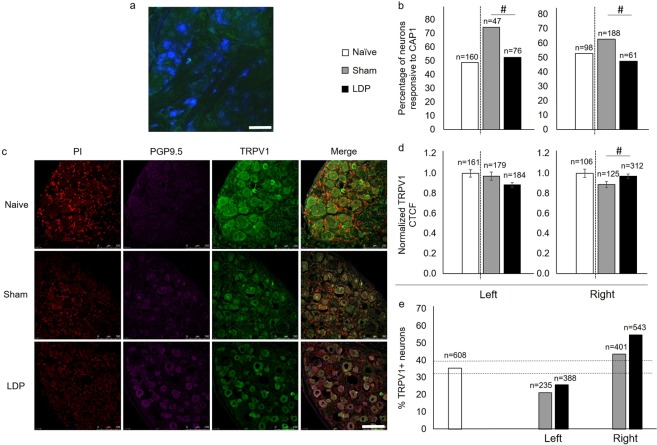
Painful IVD degeneration leads to DRG neuron remodeling and altered TRPV1 function. (**a**) A representative image of Fluoro-Gold-labeled neurons (blue) in a left L2 DRG tissue section from a naïve animal. Scale bar: 100 µm. (**b**) The percentage of neurons that responded to capsaicin *in vitro* was found to differ between sham and LDP groups bilaterally (Chi-square test of left DRG neurons and of right DRG neurons, ^#^p < 0.05). For all results, values above each bar indicate the number of neurons evaluated within each group. (**c**) Representative images of TRPV1 immunostaining of left L2 DRG tissue sections. Propidium iodide (PI). Scale bar: 100 µm. (**d**) TRPV1 fluorescence normalized to that of the naïve group was found to differ between sham and LDP groups in right DRG neurons (unpaired T-test with Welch’s correction, ^#^p < 0.05). Error bars represent standard error of the mean. (**e**) The percentage of TRPV1+ neurons was found to differ for surgical groups. Naïve group 95% confidence intervals (dotted lines): upper = 39, lower = 32.

The TRPV1 receptor has been implicated in inflammatory and neuropathic pain^25^, and NGF-dependent sensory neurons are often challenged *in vitro* with capsaicin to assess TRPV1 function^19,26^. Here, using neurons dissociated from DRGs known to innervate the L5-L6 rat IVD (Fig. 2a), we observed a decreased proportion of neurons responsive to capsaicin bilaterally in the LDP group at the chronic timepoint (Fig. 2b; left: p = 0.016, right: p = 0.036). To determine if this resulted from a decreased number of TRPV1-positive (TRPV1+) neurons or a decreased number of TRPV1 receptors following LDP injury, L1 and L2 DRGs sections were immunostained for TRPV1 (Fig. 2c) and digital micrographs were processed using ImageJ (Supplementary Fig. S4). Left TRPV1+ LDP neurons showed no difference in the immunofluorescence signal for TRPV1 (corrected total cell fluorescence, CTCF; Fig. 2d) or in the proportion of TRPV1+ neurons between groups (Fig. 2e). The right DRG neurons showed increased TRPV1 CTCF (Fig. 2d; p = 0.034) and an increased proportion of TRPV1+ neurons (Fig. 2e). This suggests that left TRPV1+ DRG neurons may have fewer TRPV1 receptors, leading to fewer capsaicin-responsive neurons in this model. The right DRG had more TRPV1+ neurons and increased TRPV1 CTCF, but still showed fewer capsaicin-responsive neurons, suggesting that these TRPV1 receptors may be functionally impaired.

**Figure 3:**
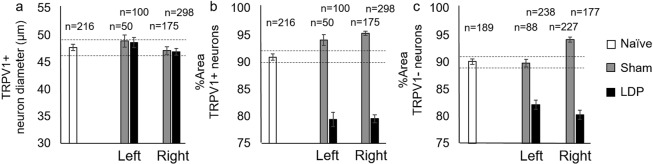
Morphologic TRPV1+ neuron characterization. (**a**) TRPV1+ DRG neuron diameter (µm) was not found to differ between sham and LDP groups. Naïve group 95% confidence intervals (dotted lines): upper = 49, lower = 46. (**b**) Cytoplasmic area in TRPV1+ neurons was found to differ between sham and LDP groups bilaterally. Naïve group 95% confidence intervals (dotted lines): upper = 92, lower = 90. (**c**) Cytoplasmic area in TRPV1- neurons was also found to differ between sham and LDP groups bilaterally. Naïve group 95% confidence intervals (dotted lines): upper = 91, lower = 89.

Investigation of morphological neuronal changes revealed no differences in maximum diameter for TRPV1+ neurons (Fig. 3a). L1 and L2 DRG neurons were measured to contain decreased cytoplasmic area in the LDP model bilaterally, independent of TRPV1+ staining (Fig. 3b,c), with the TRPV1+ neurons showing the largest reduction in cytoplasmic area. These results indicate a larger unstained nuclear region in TRPV1+ DRG neurons of the LDP group. We further investigated functional neuronal differences in this model of chronic IVD degeneration by looking at the effects of exogenous NGF on TRPV1 receptor function.

### Altered TRPV1 function was not rescued with exogenous NGF *in vitro*

**Figure 4:**
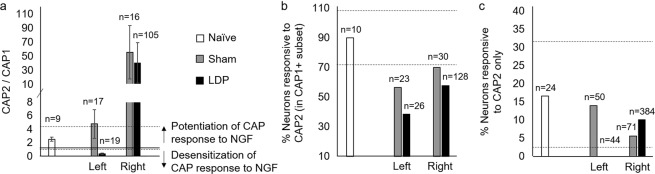
LDP injury induced DRG neuron changes that were not rescued with exogenous NGF *in vitro*. (**a**) Ratio of capsaicin responses where a ratio of <1 or >1 indicates desensitization or potentiation of the capsaicin response to NGF, respectively (solid line). Error bars represent standard error of the mean. CAP2/CAP1 was found to differ between left sham and LDP groups. Naïve group 95% confidence intervals (dotted lines): upper = 4.4, lower = 0.70. (**b**) Percentage of neurons which responded to CAP2 in CAP1-responsive subset was found to differ for surgical groups. Naïve group 95% confidence intervals (dotted lines): upper = 108, lower = 71. (**c**) Percentage of neurons which responded to CAP2, but not CAP1 was found to differ between left sham and LDP groups. Naïve group 95% confidence intervals (dotted lines): upper = 32, lower = 1.8. Values above individual bars indicate the number of neurons per treatment group.

Elevated NGF following tissue inflammation^20^ promotes ingrowth of small, nociceptive DRG neurons^10^ and can bind to TrkA to potentiate the function of TRPV1 receptors present on NGF-dependent neurons, activating the canonical TRPV1 pain pathway^27^. Therefore, we assessed functional NGF-related neuronal changes in this model via calcium imaging. Following the first application of capsaicin (CAP1), the experimental protocol continued with a period of NGF incubation and subsequent capsaicin application (CAP2) to evaluate the potentiation of TRPV1 function by NGF, a phenomenon seen in non-pathologic neurons^19,26^. The ratio of neuronal responses to capsaicin before and after NGF incubation revealed a desensitization response in the left LDP neurons (CAP2/CAP1 < 1) that fell outside the 95% confidence interval for neurons from the naïve group (Fig. 4a). All other treatment groups showed potentiation of TRPV1 function (CAP2/CAP1 > 1) (Fig. 4a). Further characterization revealed that of the neurons which responded to CAP1, fewer neurons from the surgical groups subsequently responded to CAP2, an effect which was most pronounced in neurons from the left LDP DRGs (Fig. 4b). Compared to all other groups, exogenous NGF was unable to elicit a response to capsaicin from the left LDP neurons (CAP2) for which there was no initial response to capsaicin (i.e., CAP1 ~0). (Fig. 4c). Taken together, these results indicate that TRPV1 function of left LDP neurons was not rescued by the exogenous NGF, suggesting that the expected rapid translocation and insertion of TRPV1 receptors in the cell membrane with NGF incubation^28^ did not occur.

### NGF receptor expression is remodeled in DRG neurons at the chronic timepoint

**Figure 5:**
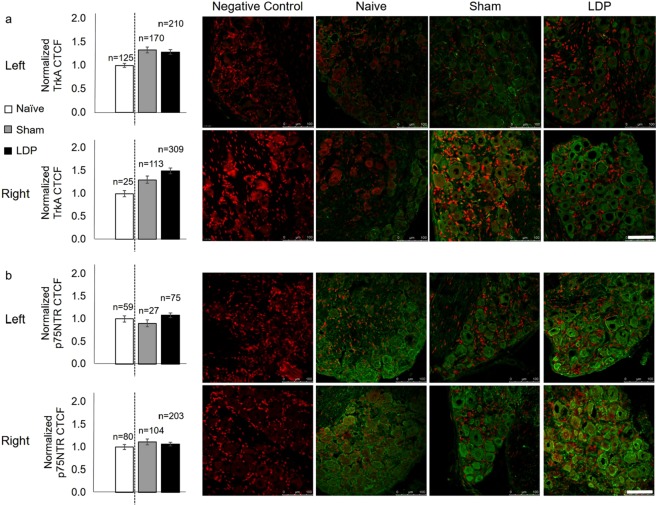
LDP injury induced trends of NGF-related neuronal changes. Although not significant, (**a**) right TrkA CTCF and (**b**) left p75NTR CTCF normalized to the naïve group were found to differ between sham and LDP groups (unpaired T-test with Welch’s correction). Error bars represent standard error of the mean. Values above individual bars indicate the number of neurons per treatment group. Scale bars: 100 µm.

NGF binding to TrkA promotes cell survival and collateral nerve sprouting, while binding to p75NTR leads to expression of additional pro-inflammatory cytokines or apoptosis^21^. The potential for NGF to differentially affect DRG neurons in the LDP model was evaluated via immunostaining for the relative expression of either receptor target. While not significant, TrkA (Fig. 5a) and p75NTR (Fig. 5b) CTCF was increased in the right (p = 0.052) and left (p = 0.057) LDP neurons, respectively, suggesting compositional changes of NGF-related receptors on neurons of innervating DRGs.

## Discussion

This study describes a clinically relevant behavioral phenotype in a rodent model of painful IVD degeneration and provides evidence of an adaptation of innervating sensory neurons following long periods of IVD pathology. As there are currently no disease modifying interventions available for chronic low back pain of this etiology and the specific mechanisms of pain generation remain unknown, this study represents a first venture into understanding the relationship between pain-related behaviors associated with IVD degeneration and the function of nerve fibers from innervating DRGs.

Injury to a single lumbar IVD is clinically relevant, as IVD lesions in humans are localized to a single lumbar disc in 62% of patients^4^. Unilateral disc puncture of one lumbar IVD revealed a bilateral behavioral phenotype characterized by reduced time spent traveling as well as decreased hind limb stride frequency compared to that of the forelimbs. Reduced open field activity has previously been shown in a model of psychological stress and chronic inflammatory pain in rats^23^. However, the current study is the first to use an overall activity measure to identify behavioral changes in a model of chronic disc degeneration at periods longer than 7 weeks of pathology development. This result has clinical relevance, as decreased general activity is well documented in patients with low back pain^29^. Decreased hind limb stride frequency is likely an adaptation due to long periods of IVD-related pain. Patients with low back pain show gait changes related to stride frequency resulting from reduced mobility of the thorax and pelvis as well as more rigid and less variable movement coordination of these components^30^. In addition, no unilateral changes were demonstrated in this model over a 20 week period of pathology development. One previous study of IVD degeneration following injury to the ventral aspect of multiple IVDs showed no unilateral behavioral changes over a 7 week study duration^12^. This result also has clinical relevance, as unilateral symptoms may be absent in lumbar discogenic pain^31^. Injury of only a single lumbar IVD and without herniation of the NP tissue may generate comparatively mild inflammation, resulting in a more subtle behavioral phenotype without unilateral changes. Therefore, the results of the current study have translational implications as similar clinically significant symptoms have been described in patients with low back pain.

Neurons from innervating DRGs in this model of painful IVD degeneration were found to be functionally different following LDP at the chronic timepoint of 20 weeks post-injury. In brief, a reduced proportion of DRG neurons responded to capsaicin bilaterally and left LDP neurons did not exhibit the expected restored response to capsaicin following NGF incubation. Left DRG neurons also demonstrated reduced TRPV1 fluorescence compared to sham DRG neurons despite a similar number of TRPV1+ neurons *in vivo*, suggesting that the isolated DRG neurons may have had fewer TRPV1 receptors or altered physiological function *in vitro*. The LDP model used a left-sided surgical approach which induced scarring on that side, but not the other. While the scarring was anatomically distant from the L1-L2 DRGs, it is possible that sensory innervation was affected by this approach. Therefore, these studies tested the differences between sham and LDP animals at 20 weeks post-surgery, but changes in left and right DRG neurons were not directly compared. It is a noteworthy limitation that we studied the entire population of neurons from the L1 and L2 DRGs and had no way here to distinguish those neurons which had true anatomic connections to the affected IVD. As such, our findings for neuronal TRPV1 function and TRPV1, TrkA and p75NTR immunopositivity were consequently confounded by a mixed population where the majority of analyzed neurons was unlikely to have anatomic connections to the degenerate IVD. Labeling neurons with a retrograde tracer or genetic marker would greatly enhance our ability to attribute changes in receptor function and presence to the affected neurons, and *in vivo* calcium imaging of the intact DRG would eliminate any phenotypic changes affecting the isolated neurons in this study. Nevertheless, an increased number of TRPV1+ neurons *in vivo* should result in a larger proportion of neurons responding to capsaicin *in vitro* despite our measurements of reduced responsiveness; our finding suggests that the TRPV1 receptors may indeed be functionally altered in the bilateral DRG neurons from the LDP injury model. This neuronal adaptation may contribute to the bilateral behavioral phenotype observed in this model.

Immunohistochemical analyses of NGF-related receptors on neurons from innervating DRGs revealed unexpected morphologic changes that may partly explain the observed altered physiology for the TRPV1 activation. Although not significant, increased p75NTR staining was demonstrated in the left LDP DRG neurons, but not right LDP DRG neurons. NGF binding to p75NTR may promote expression of additional pro-inflammatory cytokines or apoptosis^21^, and involvement of progressive inflammatory processes may be one reason for altered TRPV1 activation following capsaicin challenge in this model. Peripheral nerve injury is one model that causes DRG neurons to undergo morphologic changes, including Wallerian axonal degeneration and apoptosis. However, surviving neurons may begin to show changes associated with axonal regeneration such as nuclear and neuronal hypertrophy due to increased metabolic and transcriptional activity^32^. Indeed, our results revealed nuclear hypertrophy in bilateral LDP neurons regardless of TRPV1 positive labeling, although this change was most pronounced in the TRPV1+ neurons. These morphologic observations together with the elevated expression of p75NTR are consistent with a population of DRG neurons that responds to IVD injury and subsequent inflammation with a hypertrophic repair response.

Developing and characterizing chronic IVD degeneration in the rat allowed us to leverage the strengths of established *in vivo* models of pain disorders, while working with a larger surgical model that has been popular for successful research translation and development of therapeutic interventions. This rat model incorporated fundamental characteristics of chronic pain patients, including middle-aged and female^33,34^, as well as the ability to mitigate stress-induced behavioral changes via extensive acclimation protocols to obtain clinically relevant operant measures of pain. These strengths of the rat model remain obstacles in mouse models due to difficulties acclimating and conditioning this species^35^, as well as an animal size that limits surgical access^36^. Despite these difficulties, transgenic knockout mice provide great promise for continued studies of the mechanisms for generation of behavioral changes, pain-related sensitivities, and altered neuronal function in the LDP model of chronic IVD degeneration. Nonetheless, translational strategies for treating IVD degeneration will find value in a more complex model of pain, such as the rat.

In summary, this surgically-induced IVD injury model replicates key features of human clinical presentation of chronic discogenic pain, a stage of IVD pathology where post-surgical inflammation is reduced. This model provides a means to map sensory neuron changes to a single affected lumbar IVD and reveals a role for altered TRPV1 activation and p75NTR receptor expression in mediating the adaptive response to IVD degeneration. This study investigated the involvement of one mechanism in this model, but many other pathways associated with pain are likely contributing to the observed phenotype. Therefore, future work in this model that studies the role for NGF receptors and other pain-associated receptors such as Na_v_1.7 and related channels will be relevant for identifying appropriate therapeutic targets. The model developed here and findings provide the template for future studies to reveal the temporal onset of neuronal and behavioral changes and that establish molecular involvement through specific inhibitory assays or genetic modifications.

## Materials and Methods

### *In Vivo* Model

Female Sprague-Dawley rats (n = 36, age 15 weeks; Envigo, Indianapolis, IN) were used for this study. All experimental procedures were approved by the Institutional Animal Care and Use Committee of Washington University in St. Louis. Rats were assigned to one of three experimental groups: naïve (n = 8), sham operated (n = 14) or lumbar disc puncture (LDP, n = 14) to induce IVD degeneration. Animals were anesthetized with 1–3% isoflurane in oxygen, placed on a heating pad in a prone position and shaved and washed in the dorsal mid-thoracic to sacral area. For the LDP group (n = 14), the L5-L6 IVD was exposed via a left dorsolateral surgical approach (Supplementary Fig. S1) and a 27 gauge needle was inserted to a depth of 2 mm 10 times to puncture the IVD, with injection of 0.3 mL air each time to ensure IVD degeneration^14,37^. Muscle layers and skin were closed with 3–0 vicryl and 4-0 nylon suture (Ethicon, Somerville, NJ), respectively. Animals in the sham group (n = 14) underwent procedures for dorsolateral surgical exposure with L5-L6 IVD visualization only. Post-operative care and monitoring included sustained-release buprenorphine (1 mg/kg; ZooPharm, Laramie, WY) via subcutaneous injection lasting 72 hours, and animals were allowed free cage activity for the duration of the experiment.

### Longitudinal Measures of Behavior and Sensitivity

To minimize animal stress, each animal was acclimated to investigator handling over 7 days with subsequent acclimation to the behavioral testing equipment over 4 consecutive days. Pre-operative behavioral assessments (Table 1) were conducted and repeated longitudinally until post-operative week 20 (repeated at post-operative week 1, 4, 8, 12, 16, 18, 20 unless otherwise indicated).

#### Open Field Arena

Freely selected activity was assessed by tracking each rat’s movement within a black acrylic arena (custom-built, 60 cm × 60 cm × 60 cm) for 30 minutes during the dark portion of a 12 hour light-dark cycle; all tracking was performed without the presence of investigators or other personnel and with illumination by red light. Movement was recorded (Sony Handycam HDR-CX405, Sony Corp, New York, NY) and analyzed (EthoVision, Noldus Information Technology, Leesburg, VA) to determine % time spent traveling (Table 1). Animals were excluded from further behavioral analyses if their % time traveling fell outside of the 95% confidence interval for their group (naïve, sham, or LDP) for ≥25% of the measured timepoints (sham n = 3, LDP n = 1); these cut-off values were selected to eliminate individual behavior differences that may confound definition of a ‘group’, as the willingness of rats to participate in each test may have varied with their context and personality.

#### Static Weight-Bearing/Incapacitance

Static weight-bearing was tested by placing the animals in an acrylic chamber so that each hind limb was positioned on an individual force plate (Model BIO-SWB M, Bioseb, Vitrolles, 13845 France). The average amount of force applied by each hind limb was measured over a 3 second interval, and % weight-bearing on the left hind limb (operated side) was calculated.

#### Treadmill Gait

Locomotion was assessed using a DigiGait^TM^ treadmill and the DigiGait Imaging System^TM^ (Mouse Specifics, Quincy, MA) with a ventral-view camera recording video of paw placement through a transparent treadmill belt (naïve n = 5, sham n = 9, LDP n = 12). Gait parameters were measured at a speed of 20 cm/s (Table 1) and repeated at post-operative week 1, 4, 6, 8, 12, 16, 18, and 20.

### Euthanasia and Tissue Harvesting

At 20 weeks post-surgery, animals received an injection of euthanasia solution (150 mg/kg, IP; Med-Pharmex, Pomona, CA) and were exsanguinated via transcardial perfusion with 1X PBS (Gibco Life Technologies, Waltham, MA) by peristaltic pump. Rats were either dissected for immediate organ removal (i.e., neuron isolation), or continuously perfused with 150 mL of 4% paraformaldehyde (PFA) in PBS (i.e., histology/immunostaining).

### IVD Histology

L5-L6 IVDs were post-fixed (4% PFA, 48 hours, 4 °C), decalcified (Immunocal, StatLab, McKinney, TX, 72 hours, 4 °C), cryoprotected (30% sucrose solution, 24 h, 4 °C), embedded in OCT Tissue Tek media (Sakura Finetek Japan, Tokyo), frozen in liquid nitrogen, and stored at −80 °C. Frontal sections of each IVD (7–9 µm, Leica CM1950 cryostat, Leica Microsystems, Buffalo Grove, IL) were mounted on glass slides with spacing of 28 µm across the disc width. One section from the anterior, mid-section, and posterior aspects of each IVD was post-fixed (4% PFA), stained with 0.1% safranin-O/0.02% fast green (Sigma-Aldrich, Saint Louis, MO), and overlaid with coverslips (Permount, Fisher Scientific, Chicago, IL). Sections were imaged at 5X under brightfield (Leica TCS SPE, Leica Microsystems) and micrographs were stored for visual grading.

### DRG Cell Isolation & Calcium Imaging

L1 and L2 DRGs from each animal (naïve n = 6, sham/LDP n = 9) were acutely dissociated using previously described methods^38^ with left and right sides studied separately, unless otherwise indicated (naïve group only). Briefly, DRGs were immersed in Hank’s buffered saline solution (1X HBSS) without Ca^2+^ or Mg^2+^ (Gibco) + 10 mM HEPES (stabilizing solution) and kept on ice (<30 min). DRGs were then incubated in 45U papain (Worthington, Lakewood, NJ)/L-cysteine (Sigma) in stabilizing solution (20 min; 37 °C, 5% CO_2_), followed by 1.5 mg/mL collagenase (Sigma) in stabilizing solution (20 min; 37 °C, 5% CO_2_). Ganglia were then resuspended in culture medium (Neurobasal A with 2% B-27 Supplement, 1% penicillin/streptromycin, 1% Glutamax, 5% FBS, Gibco), and triturated with fire-polished Pasteur pipettes. The dissociated cells were filtered through a 40 μm cell strainer (Corning, Corning, NY), plated on coverslips (12 mm diameter; Fisher Scientific, Pittsburgh, PA) coated with laminin-rich matrix secreted from a rat bladder carcinoma cell line (804G)^39^, and incubated overnight in culture medium (37 °C, 5% CO_2_). To increase the likelihood of cells maintaining their *in vivo* phenotype, all experiments were performed within 24 hours of plating.

Plated cells were incubated in 3 μg/ml Fura-2 AM (45 min, Invitrogen Life Technologies, Eugene, OR) and then external solution (30 min, 130 mM NaCl, 5 mM KCl, 2 mM CaCl_2_, 1 mM MgCl_2_, 30 mM glucose, 10 mM HEPES, Sigma). Each cell-laden coverslip was placed in a perfusion chamber on an inverted microscope (Olympus Optical, Tokyo, Japan) and perfused with external solution at room temperature (2 mL/min). Images were captured with an Orca camera (Hamamatsu Photonics, Hamamatsu City, Japan) and SimplePCI Software (Hamamatsu Photonics), and regions of interest (ROIs) were manually traced and recorded over time to measure the ratio of fluorescence emission at excitation wavelengths of 357 and 380 nm.

The percentage of neurons responding to capsaicin was measured as follows: 2 minute baseline (external solution), 10 second application of KCl (50 mM) to determine live neurons, ≥6 minutes with external solution, 20 second application of capsaicin (200 nM, Sigma), and perfusion with external solution to re-establish baseline (≥8 min). Recorded tracings of individual neuron responses over time were plotted (GraphPad Prism, GraphPad Software Inc, La Jolla, CA). A ≥ 10% change from the baseline 357 nm/380 nm ratio was considered a positive response, and the number of responding neurons was recorded for each group (naïve, sham, LDP) and each side (left, right).

To test the effect of exogenous NGF on the response of TRPV1 to capsaicin, separate cell-laden coverslips were exposed to capsaicin (CAP1, 100 nM) and washed with external solution as described above, followed by 8 minutes of 2.5S NGF (50 ng/mL; Corning), 20 second application of capsaicin (CAP2, 100 nM), and washed with external solution to re-establish baseline (≥8 min). The ratio of amplitude responses to capsaicin (CAP2/CAP1), the numbers of neurons responding to CAP1 and CAP2, and numbers of neurons exhibiting a positive response to CAP2 only were recorded for each group (naïve, sham, LDP) and each side (left, right). Any cells that failed to return to baseline after responding to the application of KCl or capsaicin (CAP1) were excluded from CAP2/CAP1 analysis. Results for left and right naïve DRG neurons were pooled and the 95% confidence intervals were calculated and compared to results from the sham and LDP groups.

### DRG Immunohistochemistry

L1-L2 bilateral DRGs (n = 3 per group) were post-fixed in 4% PFA (48 hours, 4 °C), immersed in 30% sucrose (24 hours, 4 °C), embedded in OCT Tissue Tek (Sakura Finetek Japan), frozen in liquid nitrogen, and stored at −80 °C. Each L1 DRG was cryosectioned at 7 µm thickness (Leica CM1950) and sections mounted on glass slides. One section from each rat was randomly selected for immunostaining per target of interest (Supplementary Table S3). Sections were washed (0.1M Tris, pH 7.6), blocked in BSA (pH 7.6, 0.005% BSA) and 10% goat serum (1 hour), washed and blocked again (15 min) and then incubated with a primary antibody in Tris-BSA (overnight, 4 °C). Sections were then washed (0.1M Tris) and incubated with fluorescent secondary antibody (2 hours, room temperature; 5 µg/mL Alexa Fluor 488 conjugated goat anti-rabbit, Abcam, Cambridge, MA; Supplementary Table S3).

To obtain *in vivo* neuronal cell counts, one L2 DRG per treatment group was cryosectioned at 10 µm thickness and sections were mounted on glass slides; five sections separated by ≥110 µm were used to ensure that each neuron was counted only once^40^. Sections were incubated with a rabbit anti-TRPV1 primary antibody followed by a fluorescent secondary antibody as described. In the same way, sections were then incubated with a mouse anti-PGP9.5 primary antibody (5 µg/mL, Abcam), followed by incubation with a fluorescent secondary antibody (5 µg/mL Alexa Fluor 633 goat anti-mouse IgG, Abcam; Supplementary Table S3).

All sections were stained with 17% propidium iodide in Tris-BSA (PI, 10 minutes, room temperature, Sigma), mounted with ClearMount (Life Technologies), and imaged at 20X by a blinded investigator via fluorescence confocal microscopy (Leica TCS SPE). Digital micrographs of stained sections were analyzed to calculate a CTCF for each target per neuron, as follows:

$$CTCF=({I}_{cytoplasm}\,-\,{I}_{background})\,\ast \,(are{a}_{cytoplasm}),$$

where *I* is the mean fluorescence intensity per region and per cell. CTCF values for all neurons studied in the left and right side of the naive group were pooled to determine a mean and standard deviation to establish a baseline value for each immunohistochemical target.

The total number of neurons (PGP9.5+) and number of TRPV1+ neurons was used to calculate the % TRPV1+ neurons. The average maximum diameter (µm), as well as the % neuronal area which stained positive for PGP9.5 was determined for TRPV1+ cells as well as TRPV1− cells. Results for left and right naïve DRG neurons were pooled and the 95% confidence intervals were calculated and compared to results from the sham and LDP groups.

### Statistical Analysis

Data for behavioral and sensitivity parameters (Table 1) were averaged to obtain one value per animal per timepoint. Parameters measured for the naïve group were analyzed via 1-factor ANOVA with Tukey’s multiple comparisons test to test for differences amongst acute, intermediate and chronic times (α < 0.05; GraphPad). Parameters measured for sham and LDP groups were determined via 2-factor ANOVA with Sidak’s multiple comparisons test (α < 0.05; GraphPad) to test for differences between groups and amongst timepoints. For all parameters at the chronic timepoint, Cohen’s d was calculated and used to determine the effect size for differences between sham and LDP groups.

Sham and LDP group results for each calcium imaging parameter were compared against the respective 95% confidence interval for the naïve group. Values that fell within this confidence interval were considered similar to naïve, and those that fell outside were considered evidence of differences.

For each DRG immunohistochemical target, CTCF values obtained from all digital micrographs within each group were converted to one z-score distribution. CTCF values corresponding to a positive z-score (TrkA+, p75NTR+, TRPV1+, PGP9.5+) or negative z-score (TRPV1−) were used for further analyses. These CTCF values were normalized to that of the naïve group and differences between sham and LDP groups were tested via unpaired t-tests with Welch’s correction (α < 0.05; GraphPad) for left and for right DRG neurons.

## Supplementary information

Supplementary Information

## Data Availability

No data for this study has been deposited elsewhere.
