# Supplementary material for: Behavioral Compensations and Neuronal Remodeling in a Rodent Model of Chronic Intervertebral Disc Degeneration
Source: Sci Rep. 2019 Mar 6;9:3759. doi: 10.1038/s41598-019-39657-6 (PMC6403208; doi:10.1038/s41598-019-39657-6)
Supplement: Supplementary file 1 — Supplementary Information [file 41598_2019_39657_MOESM1_ESM.pdf]

Title: Behavioral Compensations and Neuronal Remodeling in a Rodent Model of Chronic Intervertebral Disc Degeneration

Authors: Elizabeth M. Leimer[1,3,4], Matthew G. Gayoso[1], Liufang Jing[1], Simon Y. Tang[2], Munish C. Gupta[2], Lori A. Setton[1,2]*

Supplementary Information:

Supplementary Materials and Methods

Validation of L1 and L2 DRG Neuron Innervation of L5-L6 IVD

Naïve female Sprague-Dawley rats (n=2, age 15 weeks; Envigo, Indianapolis, IN) were used for this validation study. This was done via the same surgical procedure as described (Supplementary Fig. S1). Once the L5-L6 IVD was exposed, Fluoro-Gold crystals (Fluorochrome; Denver, CO) were applied using 27 gauge needle inserted to a depth of 2 mm. The application site was immediately sealed with cyanoacrylate to ensure containment of the Fluoro-Gold within the IVD[9]. Muscle layers and skin were closed with 3-0 vicryl and 4-0 nylon suture (Ethicon), respectively. At 1 week post-surgery, animals received an injection of euthanasia solution (150 mg/kg, IP; Med-Pharmex) and were exsanguinated via transcardial perfusion with 1X PBS (Gibco) by peristaltic pump. Rats were then continuously perfused with 150 mL of 4% PFA in PBS. L1-L2 bilateral DRGs were post-fixed in 4% PFA (48 hours, 4°C), immersed in 30% sucrose (24 hours, 4°C), embedded in OCT Tissue Tek (Sakura Finetek Japan), frozen in liquid nitrogen, and stored at -80°C. Each DRG was cryosectioned at 7 µm thickness (Leica CM1950) and sections mounted on glass slides. Sections were mounted with ClearMount (Life Technologies) and imaged at 20X via fluorescence confocal microscopy (Leica TCS SPE).

Mechanical Sensitivity

Paw withdrawal from von Frey filaments (Stoelting, Wood Dale, IL) was tested in a pilot study to determine the threshold force for 50% paw withdrawal (50% withdrawal threshold) via Chaplan's up-down testing method[1]. During testing, animals were placed in an elevated wire-bottomed cage and each filament was placed against the paw for 6 seconds with enough applied force to bend the filament. Quick paw withdrawal or retreating from the stimulus with licking of the affected paw were considered positive responses. Data was normalized to the naïve group baseline.

Site-Specific Pressure Sensitivity

Local site pressure sensitivity of the low back was assessed using a small animal algometer (SMALGO algometer, Bioseb, Vitrolles, 13845 France). This device consists of a force gauge with a 5 mm diameter tip that was pressed directly on the skin over the dorsal aspect of the L5-L6 IVD at midline while the animal was held by an investigator. The force was increased until the animal produced an escape reaction or audible vocalization[12] to determine the applied pressure threshold.

Burrowing

Methods were adapted from Deacon[2]. Briefly, 10 cm diameter white PVC pipe was sectioned into 32 cm lengths and one end of each length was closed with a fitted black foam PVC sheet. The open end of each tube was raised 6 cm by two 8 cm bolts, each placed 2.5 cm inward from the opening and spaced 7 cm apart. Each tube was filled with 2500 g of pea-shingle gravel (diameter <1 cm, Rock City Sand & Gravel, Red Hook, NY) and placed into a rat cage (26.0 cm x 47.6 cm x 20.3 cm). Each animal was placed into an individual burrowing cage and allowed to

burrow freely for 60 minutes during the dark portion of a 12 hour light-dark cycle, and without the presence of investigators or other personnel. The behavioral testing room was illuminated by red light. The weight of displaced gravel was calculated and normalized to the amount displaced at baseline. Animals were included in the burrowing assessment analysis if they displaced at least 10% of the total gravel (250 g) at the baseline timepoint (naïve/LDP n=4, sham n=5).

Lateral Bending Maze

Pain tolerance on lateral bending was assessed using a custom-built maze with 4 alternating left and right turns, which each require extreme lateral bending of the spine for completion. At each timepoint, rats were placed at one end of the maze and allowed 2 minutes to move freely. The time each rat needed to complete 2, 4, and 8 progressive turns through the maze was recorded, as well as the total number of turns completed. Animals were included in the lateral bending maze analysis if they completed at least 8 turns within 2 minutes at the baseline timepoint (naïve n=3, sham n=6, LDP n=8).

Quantitative Analysis of DRG Immunostaining Using ImageJ Software

Neurons on the digital images of immunolabeled DRGs were identified based on the PI staining (Supplementary Fig. S4a) and manually segmented into ROIs (Supplementary Fig. S4b). ROIs were thresholded using the isodata algorithm to output a binary image (Supplementary Fig. S4c, ImageJ), and the percentage of pixels above the threshold and average intensity for all thresholded pixels (white; Supplementary Fig. S4c) determined the area fraction of positive stain in each neuron ($area_{cytoplasm}$) and mean intensity of labeling ($I_{cytoplasm}$) for the antibody of interest (TrkA, p75NTR, TRPV1).

Background regions were defined as all pixels outside all ROIs for a single field of view. Huang's fuzzy thresholding method[3] was applied to the image background to identify the average intensity of background fluorescence ($I_{background}$, Supplementary Fig. S4d).

Statistical Analyses for Histology Grading and Correlations to Behavioral Changes

Histologic images were graded to consensus by two investigators blinded to treatment group and other identifiers. Grading criteria were modified from Tam and co-workers (Supplementary Table S1)[4]. Briefly, each section was evaluated for grade of degeneration on a 0-4 scale (0: non-degenerate, 4: degenerate). The most degenerate grade in each group of sections was assigned as the grade for the entire IVD. The Wilcoxon rank-sum test was used to determine differences in the grade of degeneration between sham and LDP treatment groups ($\alpha<0.05$; GraphPad).

Correlation analysis was performed for behavioral changes against grade of IVD degeneration at the 20 week timepoint. This was done using data from each animal that completed the open field and treadmill gait assessments and the corresponding grade of degeneration for each IVD, which were ranked (GraphPad) and then correlated across all groups (CORREL function, Microsoft Excel). The Spearman's rank-order correlation coefficient (r_s) was tested for significance against the null hypothesis that $r_s=0$ ($\alpha<0.05$). For these datasets and a two-tailed analysis, significance was declared if $r_s<-0.35$ or >0.35, or $r_s<-0.39$ or >0.39, respectively (Supplementary Table S2).

Supplementary References

1. S. R. Chaplan, F. Bach, J. Pogrel, J. Chung, T. Yaksh, Quantitative assessment of tactile allodynia in the rat paw. *J. Neurosci. Methods* **53**, 55-63 (1994).

2. Deacon, Burrowing: a sensitive behavioural assay, tested in five species of laboratory rodents. *Behav. Brain Res.* **200**, 128-133 (2009).

3. L.-K. Huang, M.-J. J. Wang, Image thresholding by minimizing the measures of fuzziness. *Pattern Recognit.* **28**, 41-51 (1995).

4. V. Tam, I. Rogers, D. Chan, V. Y. Leung, K. Cheung, A comparison of intravenous and intradiscal delivery of multipotential stem cells on the healing of injured intervertebral disk. *J. Orthop. Res.* **32**, 819-825 (2014).

Supplementary Figures

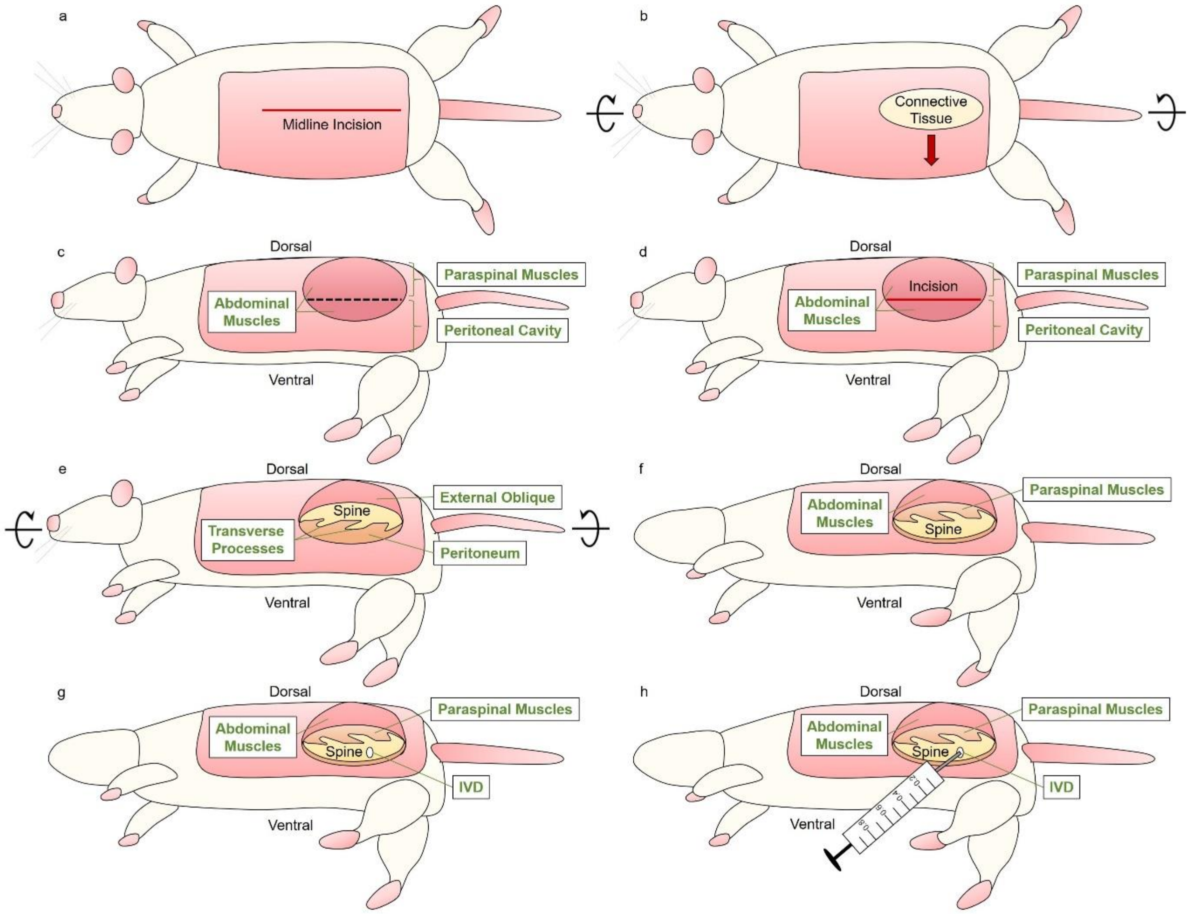

Supplementary Fig. S1. Surgical procedure to visualize and puncture the L5-L6 IVD. A 3 cm dorsal midline skin incision **(a)** was made was made, exposing the subcutaneous connective tissue **(b)**. This tissue was dissected away from the skin, and in this manner the skin of the left side of the animal was freed (B, red arrow). Then the animal was carefully rotated onto its right side (B, black arrows) and the interface of the paraspinal muscles and peritoneal cavity was palpated **(c)**. A left-sided dorsolateral incision was made through the exposed abdominal muscles at this interface **(d)**, and blunt dissection was performed to isolate the retroperitoneal space from the peritoneal cavity, and the transverse processes were identified **(e)**. Then the transverse processes were followed anteriorly and the animal was carefully rotated dorsally (e, black arrows) to allow access

to the anterolateral aspect of the spine **(f)**. The psoas muscle was separated to expose the disc space. The L5-L6 IVD was then exposed via blunt dissection **(g)** and punctured to a depth of 2 mm with a 27 gauge needle plus injection of 0.3 mL air (10x) **(h)**.

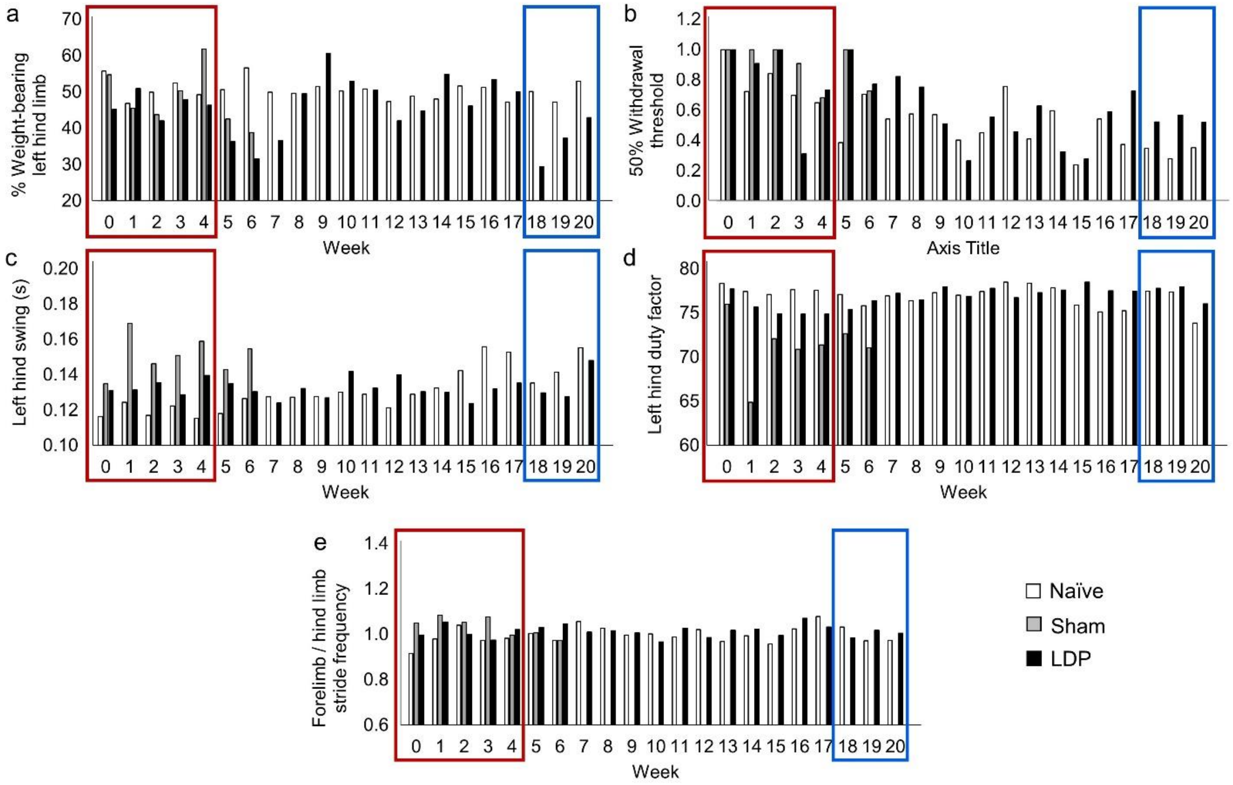

Supplementary Fig. S2. **A pilot study revealed a timeline of behavioral changes after LDP injury. (a)** Static weight-bearing for the left hind limb. **(b)** Mechanical sensitivity measured as the threshold force for 50% paw withdrawal and normalized to naïve baseline. **(c-e)** Treadmill gait parameters: left hind limb swing duration; left hind limb duty factor; forelimb vs hind limb stride frequency. Naïve: n=2, Sham: n=1, LDP: n=2. Red and blue boxes indicate the acute (0-4 weeks) and chronic (18-20 weeks) time bins, respectively, used for the full cohort study.

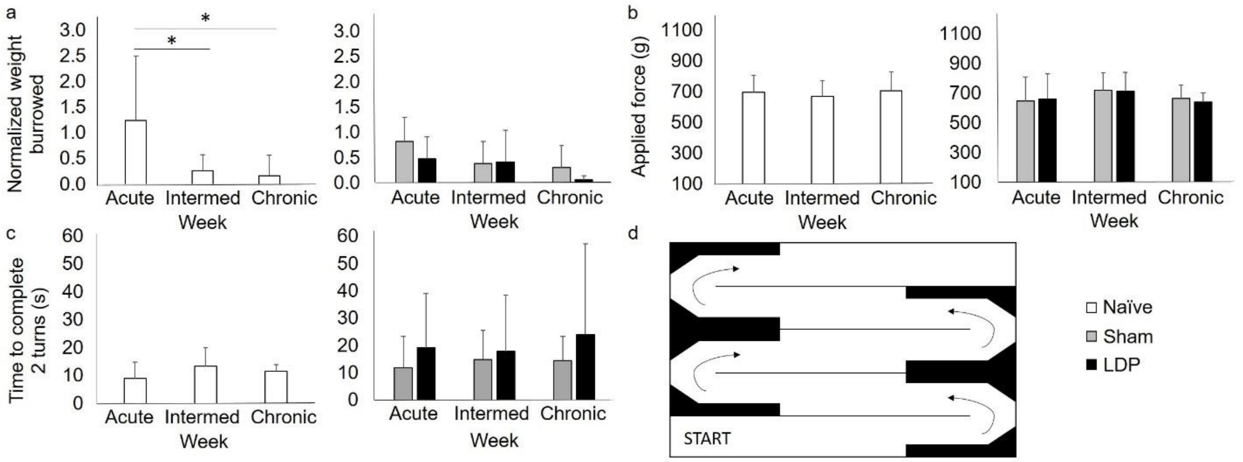

Supplementary Fig. S3. Additional behavioral assessments that demonstrated no differences between surgical groups. The amount of gravel displaced from burrowing tube normalized to pre-surgical values was shown to change over time in the naïve animals (1-way ANOVA with Tukey’s post-hoc test, *p<0.05). No differences in this parameter were detected between surgical groups (2-way ANOVA with Sidak’s post-hoc test; **a**). Force applied to dorsal spine which elicited a vocalization or escape response **(b)** and time to complete 2 progressive turns in the lateral bending maze **(c)** showed no changes over time in the naïve animals (1-way ANOVA with Tukey’s post-hoc test) or differences between surgical groups (2-way ANOVA with Sidak’s post-hoc test). All error bars represent standard deviation. **(d)** Schematic of lateral bending maze, custom-built to require extreme lateral bending of the spine to complete each turn (black arrows).

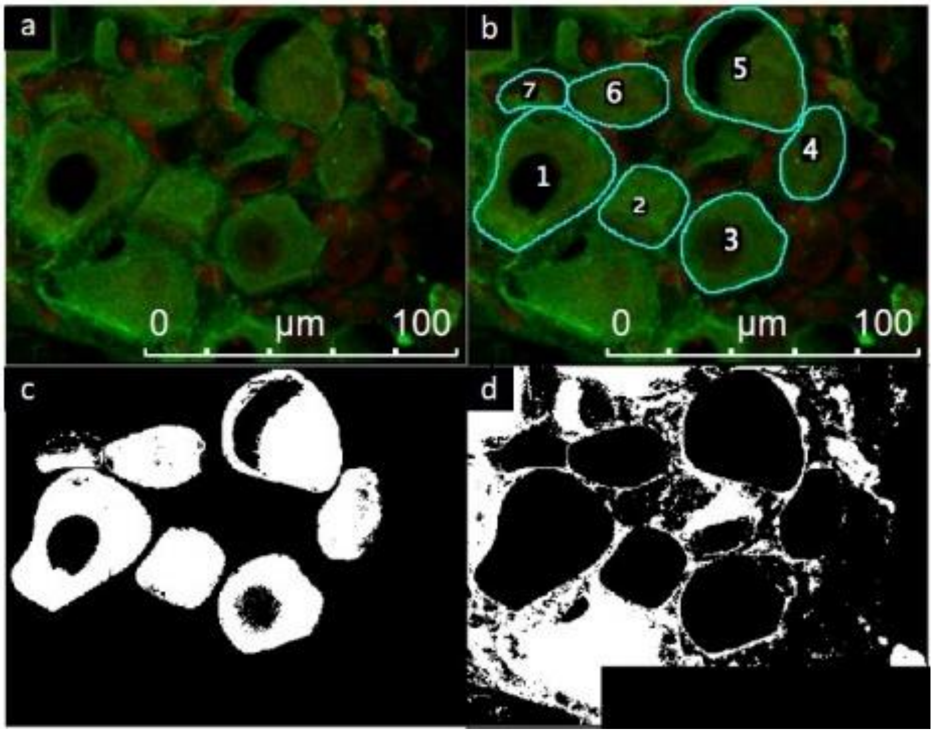

Supplementary Fig. S4. Image processing of digital micrographs of immunolabeled DRG neurons. For each antibody of interest**,** DRG neurons were analyzed to determine the intensity of labeling and the area associated with positive labeling (green; **a**). ROIs of manually segmented DRG neurons **(b)**. ROIs thresholded using the isodata algorithm **(c)**. Background regions were identified using Huang's fuzzy thresholding method **(d)**.

Supplementary Tables

Supplementary Table S1. IVD Histology Grading Criteria.

Grade	Description
0	AF lamellae clearly defined and not disrupted with clear border between AF and NP<br>NP compartment occupied predominantly by large cell mass
1	AF moderately serpentine with/without rupture<br>NP cell mass has honey comb-like structure with fragmentation into smaller cell clusters
2	AF severely serpentine with rupture. Border between AF and NP less defined<br>NP has many small cell clusters
3	AF lamellae indistinct and disorganized<br>NP has few cell clusters with/without minor cleft formation
4	AF has cleft formation with/without lamella fragmentation<br>NP compartment lost and occupied by connective tissue

Supplementary Table S2. Correlation Coefficients for Comparisons Between Behavioral and Histologic Changes.

Assessment Parameter Compared to Histologic Grade	Spearman's Correlation Coefficient	Critical Value for Spearman's Rho (r_s)
Percentage time spent traveling	-0.047	±0.35
Forelimb versus hind limb stride frequency	0.27	±0.39
Left hind limb swing duration (s)	0.11	±0.39

Supplementary Table S3. Immunohistochemistry Targets for L1 and L2 DRGs.

DRG	Target	Primary Antibody	Secondary Antibody
L1	TrkA	rabbit anti-TrkA	Alexa Fluor 488 conjugated goat anti-rabbit
	p75NTR	rabbit anti-p75NTR	Alexa Fluor 488 conjugated goat anti-rabbit
L1, L2	TRPV1	rabbit anti-TRPV1	Alexa Fluor 488 conjugated goat anti-rabbit
L2	PGP9.5	mouse anti-PGP9.5	Alexa Fluor 633 conjugated goat anti-mouse
